# Use of adjuvant chemotherapy in patients with stage III colon cancer in Puerto Rico: A population-based study

**DOI:** 10.1371/journal.pone.0194415

**Published:** 2018-03-27

**Authors:** Karen J. Ortiz-Ortiz, Guillermo Tortolero-Luna, Ruth Ríos-Motta, Alejandro Veintidós-Feliú, Robert Hunter-Mellado, Carlos R. Torres-Cintrón, Tonatiuh Suárez-Ramos, Priscilla Magno

**Affiliations:** 1 Cancer Control and Population Sciences Program, University of Puerto Rico, Comprehensive Cancer Center, San Juan, Puerto Rico; 2 Department of Health Services Administration, Graduate School of Public Health, Medical Sciences Campus, University of Puerto Rico, San Juan, Puerto Rico0; 3 Puerto Rico Central Cancer Registry, University of Puerto Rico Comprehensive Cancer Center, San Juan, Puerto Rico; 4 San Juan Bautista School of Medicine, Caguas, Puerto Rico; 5 Division of Cancer Medicine, University of Puerto Rico Comprehensive Cancer Center, San Juan, Puerto Rico; 6 College of Natural Science, University of Puerto Rico, Río Piedras Campus, San Juan, Puerto Rico; Karolinska Institutet, SWEDEN

## Abstract

**Objective:**

This study aims to examine factors associated with the use of adjuvant chemotherapy and the use of oxaliplatin after curative resection in stage III colon cancer patients and assesses the effect of their use in three-year survival.

**Methods:**

This retrospective cohort study was conducted using Puerto Rico Central Cancer Registry-Health Insurance Linkage Database. The study cohort consisted of stage III colon cancer patients with a curative surgery in the period 2008–2012. Multivariate logistic regression was used to estimate adjusted odds ratios. Kaplan-Meier methods and Cox proportional hazards models were used to assess the association between adjuvant chemotherapy and oxaliplatin use and overall survival and risk of death, respectively.

**Results:**

Overall, 75% of the study population received adjuvant chemotherapy during the study period. Factors statistically associated with receiving adjuvant chemotherapy within four months after resection included being married (adjusted odds ratio [AOR] 1.64; 95% CI 1.18–2.28; p = 0.003), and being enrolled in Medicare (AOR 1.68; 95% CI: 1.03–2.75; p = 0.039) or Medicaid and Medicare dual eligible (AOR 1.66; 95% CI: 1.06–2.60; p = 0.028). However, patients aged ≥70 years were less likely to receive adjuvant chemotherapy (AOR 0.22; 95%CI 0.14–0.36; p<0.001).

**Discussion:**

We observed a significant reduction in mortality in adjuvant chemotherapy treated patients. Similarly, patients <70 years treated with oxaliplatin had significantly lower risk of death than those who did not, although for patients ≥70 years no statistical significance was achieved. Future studies should assess effective interventions to reduce barriers to access guideline-based recommended colon cancer treatment.

## Introduction

Adherence to recommended evidence-based guidelines is crucial to improve health outcomes. Surgical resection is the standard treatment for patients with colon and rectal cancer. For patients with stage III colon cancer, the National Comprehensive Cancer Network (NCCN) guidelines recommends surgical resection and adjuvant chemotherapy (ACT) [1]. A robust body of evidence indicates that using ACT decreases the risk of recurrence and improves survival among stage III colon cancer patients [2–10]. It has also been demonstrated that including oxaliplatin in the drug combination has significantly improved the disease-free survival and overall survival, particularly in patients <70 years old [3,11–14]. The NCCN guidelines recommend the use of FOLFOX (fluorouracil with modulating leucovorin (FU) and oxaliplatin) and CAPEOX (capecitabine and oxaliplatin) as the preferred ACT regimen [1]. However, the benefit of oxaliplatin in patients age 70 and older has not been confirmed [1,3,8,9]. Nevertheless, even with the proven benefits of ACT, several studies have shown differences in the use of ACT by age, race/ethnicity, type of insurance coverage and socioeconomic status [2,10,11,15–21].

Puerto Rico, a territory of the United States (US), has a population of approximately 3.4 million, primarily of Hispanic origin (98%) and over 90% of the population has health insurance coverage. In Puerto Rico, approximately 49% of the population (19% in the U.S.) receives health services through Medicaid/CHIP (Government Health Plan), 34% from private insurance (59% in the U.S.), 11% from Medicare/Military (10% in the U.S.), and 6% are uninsured (12% in the U.S.) [22].

In spite of the high percentage of the Puerto Rico population with access to health insurance, differences in health outcomes remain. For example, our group reported that colorectal cancer patients covered by the Government Health Plan (Medicaid) are diagnosed in more advanced stages and have poorer survival rate than those with private health insurance [23].

Knowing and understanding the barriers to access to evidence-based health care is fundamental to address these disparities and to reduce the social and economic burden of colon cancer in Puerto Rico. To our knowledge, this is the first study in Puerto Rico exploring the pattern of using guideline-recommended treatment for stage III colon cancer, its impact on health outcomes, and the factors associated with receiving evidence-based treatment. This study aims to examine the following questions: What is the pattern of using guideline-recommended treatment after curative resection of stage III colon cancer? What are the patient and clinical characteristics associated with receiving ACT with or without oxaliplatin after curative resection of stage III colon cancer patients? Is 3-year survival rate of patients that received ACT with or without oxaliplatin different to those who did not?

## Materials and methods

### Data sources

This retrospective cohort study used the Puerto Rico Central Cancer Registry- Health Insurance Linkage Database (PRCCR-HILD) to identify colon cancer cases diagnosed during the period 2008–2012. The PRCCR-HILD is a resource developed by the PRCCR to conduct cancer care delivery research examining patterns, quality, and outcomes of cancer care across the cancer care continuum in the island. PRCCR law allows the gathering of insurance companies’ administrative claims. The PRCCR-HILD links the PRCCR database to the claims database received from the major health insurance companies in the island including Medicaid, Medicare, and private insured population. Currently, the PRCCR-HILD includes over 180 million claims for approximately 82% of all the cases diagnosed between 2008 and 2014. For the proposed study period (2008–2012), the PRCCR-HILD contains all the insurance claims generated for over 80% of all cancer patients and 85% of all colon cancer patients reported during this period. PRCCR provided information regarding patient’s sociodemographic characteristics, diagnostic variables, tumor variables, and follow-up information. Whereas, treatment information including date of service, procedure codes (CPT/HCPS), National Drug Codes (NDC), diagnostic codes (ICD9-CM and ICD10-CM format), patient information, service provider information, cost of services, and enrollment files, among others, was obtained from the PRCCR-HILD database. PRCCR cases were matched to the health insurance companies data utilization files using a deterministic matching similar to the algorithm used by SEER-Medicare [24]. In addition, PRCCR receives mortality files from the Puerto Rico Demographic Registry, which covers the entire population. Annually, the PRCCR database is linked with mortality files.

### Study population

The preliminary study cohort consisted of patients with adenocarcinoma of colon (excluding appendix and rectum) diagnosed during the period 2008–2012, who fulfilled the American Joint Cancer Committee criteria for stage III and who have had a colon resection. Given that follow-up information was available until December 2015, we included patients diagnosed until 2012 to give all the patients a minimum follow-up time of 3 years. We included only residents of Puerto Rico with a first cancer diagnosis. The study population was limited to those with complete claims information. Patients who died within 30 days of surgery were excluded. Table 1 shows selection criteria including case exclusion.

**Table 1:** Cohort selection criteria for patients with stage III colon cancer diagnosed during 2008–2012.

Exclusion Criteria	Exclusions	Remaining	%
Assessed for eligibility	-	1,638	100.0
Non-resident of PR at diagnosis	6	1,632	99.6
2^nd^ and later primary diagnosis of cancer	205	1,427	87.1
Patient data not available in claims database or insurance gaps	289	1,135	69.3
Non colon resection	16	1,122	68.5
Death within 30 days of surgery	87	1,035	63.2

### Treatment measurements

To examine factors associated with the use of ACT and the use of oxaliplatin we obtained information from claims data, including pharmacy claims. We included chemotherapy agents that are recommended for colon cancer stage III (fluororacil, leucovorin, capecitabine, oxaliplatin or levoleucovorin) and that were received within four months after the colon surgery. Chemotherapy and surgery codes are documented in the S1 Table. A dichotomous variable was generated to indicate if the patient received ACT. Patients with no claims for recommended chemotherapy agents within four months after the colon surgery were categorized into the group who did not receive ACT. Patients who received ACT were classified into those with oxaliplatin agents and those with no oxaliplatin agents.

### Covariates

Patient characteristics included: age at diagnosis (grouped into three categories: <60, 60–69, and ≥70 years), sex, marital status (unmarried, married, and unknown), area of residence at the time of diagnosis (based on Puerto Rico Health Insurance Administration geographic service regions), and comorbidity. Comorbidity was assessed using the Klabunde modification of the Charlson Comorbidity Index (CCI) [25]. For some patients CCI could not be calculated because the claims before the cancer diagnosis were not available. Tumor variables evaluated were year of diagnosis, primary tumor location (distal, proximal, and overlapping/unknown), and histologic grade (well/moderately differentiated, poorly/undifferentiated, and unknown).

### Statistical analyses

We performed a Chi-square or Fisher's exact test to assess the associations between categorical independent variables and the use of ACT or oxaliplatin. Multivariate logistic regression models were used to estimate the adjusted odds ratio (AOR), and their 95% confidence intervals (CI). Variables with a p-value <0.20 in the bivariate analysis were included in the multivariable logistic regression models to adjust for potential confounding variables. The likelihood ratio test was used to assess the significance of interaction terms.

We used the Kaplan–Meier method to compare the 3-year survival curves among patients who received ACT with those who did not and patients who received oxaliplatin with those who received ACT but not oxaliplatin. Log rank test and Cox were used to evaluate statistical differences between curves. Multivariate Cox regression analysis was performed to estimate the adjusted effect of receiving ACT or oxaliplatin on 3-year overall survival. Propensity scores were calculated to take into account the likelihood of receiving ACT or oxaliplatin. This variable was categorized into deciles and was included as a covariate in the Cox regression models. The proportionality assumption was evaluated using Schoenfeld residuals. To address the no-compliance with the proportionality assumption in our Cox models we stratified by age-groups (<70 years and > = 70 years). All statistical analyses were performed using the Stata/SE statistical software (version 14.2, College Station, TX).

### Ethics statement

The study was summited and approved by the Institutional Review Board of the University of Puerto Rico, Medical Sciences Campus, San Juan, Puerto Rico. The analysis included secondary data with de-identified patient records. All information from patients was held in strict confidence. Patient ID fields within the PRCCR database are encrypted using a bijective function based on prime numbers. Both encrypt and decrypt functions are stored in a secure server. Only the research team of this study had access to patient information, and only information relevant to this study was examined.

## Results

A total of 1,035 patients with stage III colon cancer were eligible for the study. Table 2 shows the study population characteristics by ACT category (receiving/no-receiving). During the study period, 75% of the study population received ACT. The use of ACT varied significantly by age, marital status, and type of insurance coverage (p<0.05). Among patients who did not receive ACT, 64% were 70 years of age and older, 50% were unmarried, and 36% had dual Medicare and Medicaid insurance. There were no statistically significant differences in the use of ACT by sex, CCI, area of residence, year of diagnosis, primary tumor location, and tumor grade (p>0.05).

**Table 2:** Characteristics of stage III colon cancer patients by adjuvant chemotherapy (ACT) status.

Characteristics	Total study population *N (%)*	ACT (n = 777) *N (%)*	No ACT (n = 258) *N (%)*	P-value*
**Study population**	1,035 (100)	777 (75)	258 (25)	
**Age group (years)**				<0.0001
<60	282 (27)	242 (31)	40 (16)	
60–69	333 (32)	281 (36)	52 (20)	
70+	420 (41)	254 (33)	166 (64)	
**Sex**				0.190
Male	538 (52)	413 (53)	125 (48)	
Female	497 (48)	364 (47)	133 (52)	
**Marital status**				<0.0001
Unmarried	401 (39)	273 (35)	128 (50)	
Married	575 (56)	457 (59)	118 (46)	
Unknown	59 (6)	47 (6)	12 (5)	
**CCI**				0.090
0	575 (56)	445 (57)	130 (50)	
1	197 (19)	142 (18)	55 (21)	
≥2	198 (19)	138 (18)	60 (23)	
Unknown	65 (6)	52 (7)	13 (5)	
**Type of insurance coverage**				0.001
Medicaid	274 (26)	209 (27)	65 (25)	
Private	230 (22)	194 (25)	36 (14)	
Medicare/Medicaid	319 (31)	227 (29)	92 (36)	
Medicare	212 (21)	147 (19)	65 (25)	
**Region**				0.092
Metro-North	185 (18)	140 (18)	45 (17)	
San Juan	98 (9)	71 (9)	27 (10)	
East	167 (16)	133 (17)	34 (13)	
North	128 (12)	102 (13)	26 (10)	
Northeast	122 (12)	79 (10)	43 (17)	
Southeast	98 (9)	73 (9)	25 (10)	
Southwest	73 (7)	59 (8)	14 (5)	
West	164 (16)	120 (15)	44 (17)	
**Diagnosis year**				0.321
2008	182 (18)	135 (17)	47 (18)	
2009	193 (19)	151 (19)	42 (16)	
2010	223 (22)	171 (22)	52 (20)	
2011	202 (20)	155 (20)	47 (18)	
2012	235 (23)	165 (21)	70 (27)	
**Primary tumor location**				0.138
Distal	435 (42)	340 (44)	95 (37)	
Proximal	589 (57)	429 (55)	160 (62)	
Other	11 (1)	8 (1)	3 (1)	
**Tumor grade**				0.164
Well/moderately differentiated	839 (81)	639 (82)	200 (78)	
Poorly/undifferentiated	116 (11)	79 (10)	37 (14)	
Unknown	80 (8)	59 (8)	21 (8)	

* p values were calculated using chi-squared test or Fisher's exact test.

Table 3 compares the characteristics of subjects receiving oxaliplatin to those no-receiving oxaliplatin as part of their ACT. Among patients receiving ACT, 76% received oxaliplatin as a part of their treatment. Receiving oxaliplatin differed statistically by age-groups, type of insurance coverage, and year of diagnosis. Patients <70 years, dual Medicare/Medicaid, and those diagnosed in 2008 were less likely to receive oxaliplatin (p<0.05). No significant differences were observed by sex (p = 0.412), marital status (p = 0.102), CCI (p = 0.208), region (p = 0.254), primary tumor location (p = 0.079), and tumor grade (p = 0.164).

**Table 3:** Characteristics of stage III colon cancer patients who used ACT by oxaliplatin Receipt status.

Characteristics	Patients Receiving ACT N (%)	Oxaliplatin N (%)	No Oxaliplatin N (%)	P-value
Study Population	777 (100)	591 (76)	186 (24)	
**Age group (years)**				<0.0001
<60	242 (31)	203 (34)	39 (21)	
60–69	281 (36)	231 (39)	50 (27)	
70+	254 (33)	157 (27)	97 (52)	
**Sex**				0.412
Male	413 (53)	319 (54)	94 (51)	
Female	364 (47)	272 (46)	92 (50)	
**Marital Status**				0.102
Unmarried	273 (35)	206 (35)	67 (36)	
Married	457 (59)	355 (60)	102 (55)	
Unknown	47 (6)	30 (5)	17 (9)	
**CCI**				0.208
0	445 (57)	338 (57)	107 (58)	
1	142 (18)	114 (19)	28 (15)	
≥2	138 (18)	97 (16)	41 (22)	
Unknown	52 (7)	42 (5)	10 (5)	
**Type of insurance coverage**				<0.0001
Medicaid	194 (25)	167 (28)	27 (15)	
Private	209 (27)	168 (28)	41 (22)	
Medicare/Medicaid	227 (19)	161 (27)	66 (36)	
Medicare	147 (19)	95 (16)	52 (28)	
**Region**				0.254
Metro-North	140 (18)	104 (18)	36 (19)	
San Juan	71 (9)	58 (10)	13 (7)	
East	133 (17)	95 (16)	38 (20)	
North	102 (13)	72 (12)	30 (16)	
Northeast	79 (10)	59 (10)	20 (11)	
Southeast	73 (9)	62 (10)	11 (6)	
Southwest	59 (8)	45 (8)	14 (8)	
West	120 (15)	96 (16)	24 (13)	
**Diagnosis year**				0.006
2008	135 (17)	88 (15)	47 (25)	
2009	151 (19)	115 (20)	36 (19)	
2010	171 (22)	136 (23)	35 (19)	
2011	155 (20)	115 (20)	40 (22)	
2012	165 (21)	137 (23)	28 (15)	
**Primary tumor location**				0.079
Distal	340 (44)	267 (45)	73 (39)	
Proximal	429 (55)	316 (53)	113 (61)	
Other	8 (1)	8 (1)	0 (0)	
**Tumor grade**				0.164
Well/moderately differentiated	839 (81)	639 (82)	200 (78)	
Poorly/undifferentiated	116 (11)	79 (10)	37 (14)	
Unknown	80 (8)	59 (8)	21 (8)	

### Use of ACT

Results from the multivariate logistic model for ACT use are shown in Table 4. Compared to patients aged <60 years, patients aged ≥70 years were less likely to receive ACT (AOR 0.22; 95% CI 0.13–0.35; p<0.001). Whereas, compared with patients enrolled in Medicaid, patients enrolled in Medicare (AOR 1.68; 95% CI: 1.03–2.75; p = 0.039) and those with dual enrollment for Medicare and Medicaid (AOR 1.66; 95% CI: 1.06–2.60; p = 0.028) were more likely to receive ACT. Likewise, patients enrolled in private insurance were more likely to receive ACT than patients enrolled in Medicaid; however, this association did not reach statistical significance (AOR 1.57; 95% CI: 0.95–2.58; p  =  0.076). In addition, married patients were more likely to receive ACT (AOR 1.64; 95% CI 1.18–2.28; p = 0.003) and those living in the Northeast region of the island were less likely to received ACT (AOR 0.58; 95% CI 0.34–0.99; p = 0.046).

**Table 4:** Predictors of Receipt ACT.

Characteristics	AOR (95% CI)	P-value
**Age group (years)**		
<60	1.00	
60–69	0.84 (0.52–1.36)	0.489
70+	0.22 (0.13–0.35)	<0.0001
**Sex**		
Male	1.00	
Female	1.05 (0.76–1.45)	0.764
**Marital status**		
Unmarried	1.00	
Married	1.64 (1.18–2.28)	0.003
Unknown	1.92 (0.94–3.89)	0.072
**CCI**		
0	1.00	
1	0.68 (0.46–1.02)	0.063
≥2	0.81 (0.54–1.20)	0.288
Unknown	1.12 (0.56–2.24)	0.740
**Type of insurance coverage**		
Medicaid	1.00	
Private	1.57 (0.95–2.58)	0.076
Medicare/Medicaid	1.66 (1.06–2.60)	0.028
Medicare	1.68 (1.03–2.75)	0.039
**Region**		
Metro-North	1.00	
San Juan	0.95 (0.52–1.71)	0.853
East	1.45 (0.85–2.49)	0.176
North	1.32 (0.73–2.37)	0.358
Northeast	0.58 (0.34–0.99)	0.046
Southeast	1.03 (0.56–1.90)	0.912
Southwest	1.43 (0.7–2.94)	0.325
West	1.01 (0.6–1.71)	0.959
**Primary tumor location**		
Distal	1.00	
Proximal	0.95 (0.69–1.31)	0.744
Other	0.46 (0.11–1.84)	0.269
**Tumor grade**		
Well/moderately differentiated	1.00	
Poorly/undifferentiated	0.74 (0.46–1.16)	0.190
Unknown	1.07 (0.61–1.89)	0.804

#### Use of oxaliplatin

Results from the multivariate logistic model for oxaliplatin use are shown in Table 5. Compared with patients aged <60 years, patients aged ≥70 years were less likely to receive oxaliplatin (AOR: 0.45; 95%CI: 0.26–0.78; p = 0.005); whereas, patients diagnosed after 2008 were more likely to receive oxaliplatin (p<0.05). No significant associations were observed with marital status, CCI, type of insurance coverage, primary tumor location, and tumor grade.

**Table 5:** Predictors of Receipt oxaliplatin.

Characteristics	AOR (95% CI)	P-value
**Age group (years)**		
<60	1.00	
60–69	1.03 (0.62–1.71)	0.921
70+	0.45 (0.26–0.78)	0.005
**Marital status**		
Unmarried	1.00	
Married	1.14 (0.78–1.67)	0.493
Unknown	0.53 (0.27–1.07)	0.078
**CCI**		
0	1.00	
1	1.29 (0.79–2.13)	0.311
≥2	0.84 (0.53–1.35)	0.482
Unknown	1.36 (0.63–2.96)	0.432
**Type of insurance coverage**		
Medicaid	1.00	
Private	1.51 (0.87–2.63)	0.147
Medicare/Medicaid	0.81 (0.48–1.37)	0.433
Medicare	0.63 (0.35–1.12)	0.114
**Diagnosis year**		
2008	1.00	
2009	1.99 (1.15–3.47)	0.015
2010	2.29 (1.32–3.95)	0.003
2011	1.81 (1.05–3.11)	0.031
2012	3.11 (1.74–5.55)	<0.0001
**Primary tumor location**		
Distal	1.00	
Proximal	0.91 (0.63–1.32)	0.634
Other		
**Tumor grade**		
Well/moderately differentiated	1.00	
Poorly/undifferentiated	1.68 (0.89–3.16)	0.107
Unknown	0.62 (0.33–1.15)	0.131

### Overall survival by age group

**Fig 1:**
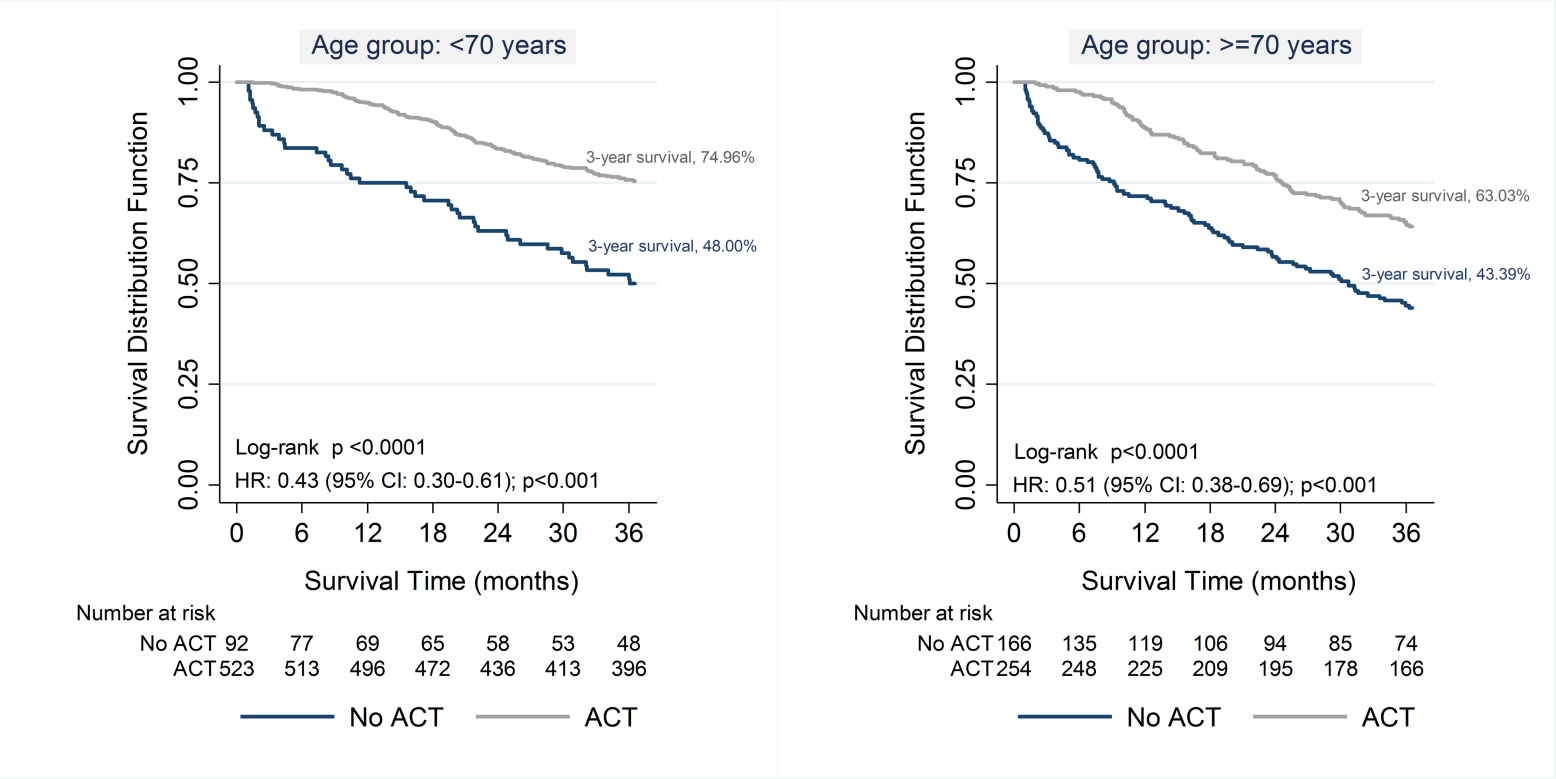
Three-year overall survival according to the Receipt of ACT stratified by age group (<70 years and ≥70 years) ACT = adjuvant chemotherapy.

Fig 1 shows the effect of ACT on 3-year overall survival stratified by age-group (<70 years and ≥70 years). For both age groups the overall 3-year survival was statistically higher (Log Rank Test p<0.05) among ACT treated patients (patients aged <70 years: 75% vs. 48%; patients aged ≥70: 63% vs. 43%, respectively). For both age-groups, patients treated with ACT had significantly lower risk of dying (aged<70 years: HR: 0.43; 95% CI: 0.30–0.61; p<0.001; and aged ≥70 years: HR: 0.51; 95% CI: 0.38–0.69; p<0.001).

**Fig 2:**
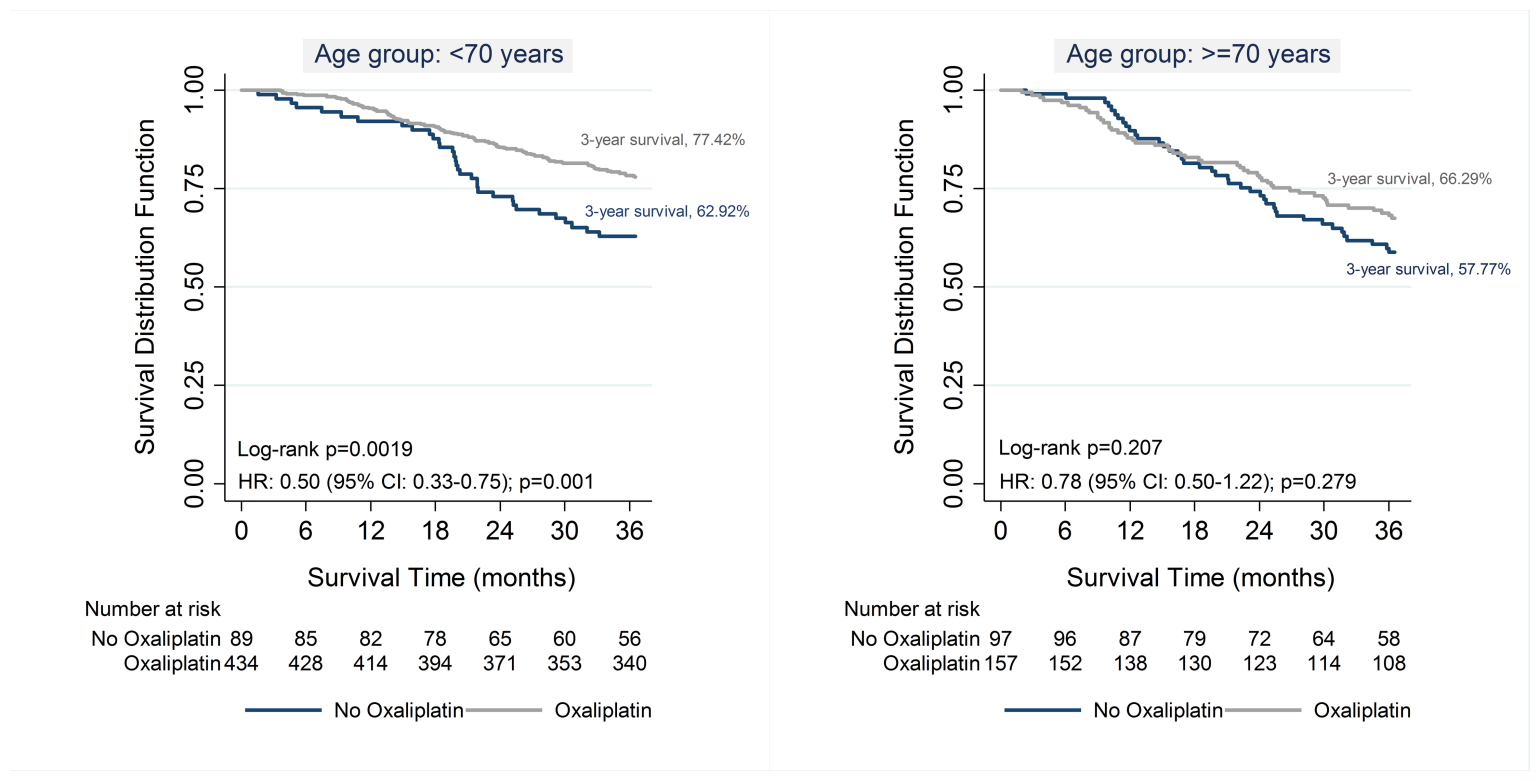
Three-year overall survival according to the Receipt of oxaliplatin among patients receiving ACT by age group (<70 years and ≥70 years).

Use of oxaliplatin showed a survival benefit only among patients aged <70 years (77% vs. 63%; Rank test p<0.05) but not among patients aged ≥70 years (66% vs. 58%; Log Rank test p = 0.2). Patients aged <70 years receiving oxaliplatin had a significant lower risk of dying (HR: 0.50; 95% CI: 0.33–0.75; p = 0.001); whereas, no statistical significance difference was achieved among patients aged ≥70 years (HR: 0.78; 95% CI: 0.50–1.22; p = 0.279) (Fig 2).

## Discussion

Understanding the pattern of care and barriers for the use of evidence-based guidelines is important to increase access to quality care, improve health outcomes, and reduce health disparities. This study examines, for the first time, the patterns of use of ACT and oxaliplatin for stage III colon cancer in Puerto Rico, the factors associated with the use of these guideline-recommended treatments in the island, and their impact on 3-year overall survival and risk of death. The results of this study can help to improve the quality of care for stage III colon cancer and address disparities in cancer care delivery and health outcomes.

Overall, 75% of the study population received ACT. Although comparable with previous population based studies, the percentage patients receiving ACT was higher than the reported in recent studies [2,4,10,15,19,20]. This difference might be attributable to the high health insurance coverage in Puerto Rico (96.3%) [26] and the adherence of health professionals in Puerto Rico to evidence-based treatment guidelines recommended. A recent study comparing coverage and access to care in Puerto Rico and the mainland US found that, despite the economic conditions in Puerto Rico, it has better coverage and access to care indicators [27]. The authors of this study also point out that these results are consistent with the fact that states with more unrestrictive Medicaid eligibility have better access to care [27]. On the other hand, this difference might also be explained by differences in data source, study population, and period of observation.

Consistent with previous studies [2–4,10,16,28–30], our data showed that patients with stage III colon cancer receiving ACT had a statistically significant reduction in mortality compared with those no receiving ACT. Our data also showed that patients aged ≥70 years, Medicaid enrollees, unmarried patients, and patients who resided in the northeast region of the Island at the time of diagnosis were less likely to receive ACT. Patients’ age has been previously reported to be a strong predictor for no receiving ACT [10,15,16,19–21,31], despite the fact that several studies, including the present study, have shown a benefit of ACT in older patients [2–4,16,32,33]. Patients aged ≥70 years who received ACT had better survival and lower risk of death compared to those no receiving ACT.

Compared to patients enrolled in Medicaid, those enrolled in Medicare and dual Medicaid and Medicare were more likely to receive ACT. These findings are consistent with previous studies [10,30,34] and with studies conducted in Puerto Rico [35–38]. Our group showed that cancer patients enrolled in the Puerto Rico’s Government Health Plan have poorer health outcomes and less access to guideline-recommended treatment compared to those enrolled in other health insurances coverage [35–38]. Even when Puerto Rico has a high insurance coverage, the fact that Medicaid patients are less likely to receive ACT could suggest that Medicaid patients may not be receiving guideline-recommended treatment for stage III colon cancer. It has been suggested that other unmeasured socioeconomic characteristics may partially explain the observed differences found in cancer care delivery for stage III colon cancer [38].

Similar to previous studies [11,17,19], we found that patients diagnosed in later years were more likely to receive oxaliplatin. In fact, when we stratified by age groups we observed the same pattern (data not shown). This finding could be attributable to the progressively adoption of oxaliplatin as a part of adjuvant chemotherapy among clinicians. In 2004, oxaliplatin was approved for use in patients with stage III colon cancer. After this event, the oxaliplatin-based regimens became the preferred ACT regimens for patients with resected stage III colon cancer [11,17,19,39].Large population-based studies using SEER data found that unmarried patients are at significantly higher risk of presenting metastatic cancer, undertreatment, and death resulting from their cancer [40–42]. Consistent with previous studies, our findings showed that unmarried patients had lower odds of receiving ACT compared to married patients. This association might be explained by other factors such as age, income, type of health insurance, and social support, among others. We also found that patients living in the Northeast region at the time of diagnosis were less likely to receive ACT. A plausible explanation of the underutilization of ACT in this region could be attributable to the access to treatment in the island-municipalities of Vieques and Culebra. Further analyses are warranted to asses possible factors associated with difference in geographic access to guideline-recommend treatment.

There was not a statistically significant benefit of receiving oxaliplatin among patients aged ≥70 years (HR = 0.78; 95% CI: 0.50–1.22) compared to younger patients. As we expected, patients aged ≥70 years were less likely to receive oxaliplatin since current NCCN guidelines do not recommend its use in these patients. The use of oxaliplatin in this population should be evaluated taking into account the toxicity and the patient’s characteristics and preferences.

The findings in this study are subject to some limitations. First, we limited our analysis to patients with health insurance coverage and that had claims data. Therefore, caution must be taken when generalizing these results. Second, due to the nature of claims data, as they are collected for reimbursement purposes, have inherent limitations when used in research studies. Third, we only evaluated the use of ACT if the patient had a claim with ACT, but we are not able to assess patient’s adherence to recommended treatment. Also, it is important to note that, by definition and according to guidelines, only those patients receiving ACT within four months after colon surgery were considered to have received ACT. Finally, another possible limitation is that we did not evaluate patient’s preferences. For example, we did not evaluate if the patient refused the chemotherapy treatment. Nevertheless, results of this study are consistent with other studies and confirms the viability of the use of the PRCCR-HILD database in cancer care delivery research.

In conclusion, similar to other populations, we found that only 25% of the stage III colon cancer do not receive guideline-recommended treatment. In addition, consistent with previous studies, we identified disparities in the use of ACT in the Puerto Rico population that need to be addressed. Considering that combining men and women colorectal cancer is the leading cause of cancer death in Puerto Rico, this study raises the need for further assess the pattern of cancer treatment in the island, identify barriers to access guideline-recommend cancer treatment, and the development and implementation of effective multilevel interventions to reduce barriers to guideline-based recommended treatment for stage III colon cancer patients. This is particularly relevant given the economic and financial crisis in Puerto Rico and uncertainty in Medicaid funding that might leave thousands of patients without access to care. Furthermore, this problem will be compounded by the prevailing migration of healthcare professional to the U.S. mainland.

## Supporting information

S1 Table Treatment associated claims codes. (PDF)
