# Supplementary material for: Use of adjuvant chemotherapy in patients with stage III colon cancer in Puerto Rico: A population-based study
Source: PLoS One. 2018 Mar 27;13(3):e0194415. doi: 10.1371/journal.pone.0194415 (PMC5870969; doi:10.1371/journal.pone.0194415)
Supplement: S1 Table — (PDF) [file pone.0194415.s001.pdf]

S1 Table: Treatment Associated Claims Codes

Procedure	CPT/HCPCS	ICD-9-CM procedure codes	NDC
Surgery resection	44140-44160, 44204-44213	45.71-45.76, 45.79, 45.8	
Chemotherapy	J0640, J0641, J9190, S3722, J9263, J8520, J8521		00054449613, 00054449625, 00054449705, 00054449710, 00054449810, 00054449911, 00054849619, 00143955201, 00143955301, 00143955401, 00143955501, 00143955801, 00555048401, 00555048402, 00555048405, 00555048504, 00555048527, 00591413054, 00703514001, 00703514501, 00703514591, 00781320194, 25021081310, 25021081366, 25021081430, 25021081467, 25021081530, 25021081567, 25021081630, 25021081667, 45963076257, 51079058106, 51079058205, 54868331000, 54868331001, 54868331002, 54868331003, 54868331004, 54868591500, 60687022794, 63323071050, 63323071059, 63323071100, 64661065030, 64661071130, 67457060020, 67457060130, 68001028536, 68001028537, 68001028540, 68001028638, 68152010100, 68152010201, 68152010202, 70121109901, 70260042601, 70260042701, 70260042801, 70260042901, 70260043001, 70260048001, 70271042601, 70271042701, 70271042801, 70271042901, 70271043001, 70271048001, 00069016902, 00069017302, 00069017401, 00069017601, 00703301513, 00703301812, 00703301912, 16729027611, 16729027638, 16729027667, 16729027668, 25021021598, 25021021599, 43547025801, 43547025901, 51672406201, 51672406301, 63323011710, 63323011718, 63323011719, 63323011720, 63323011728, 63323011751, 63323011758, 63323011759, 63323011761, 63323011768, 63323011769, 66758004403, 66758005401, 66758005402, 68001026627, 68001026630, 68001026631, 68001026632, 00024059010, 00024059120, 00069006701, 00069007001, 00069007401, 00069101001, 00703398501, 00703398601, 00781331570, 00781331780, 00781931570, 00781931780, 00955172510, 00955172720, 00955173110, 00955173320, 12516059204, 25021021120, 25021021250, 25021023310, 25021023320, 45963061153, 45963061159, 47335004640, 47335004740, 47335017640, 47335017840, 60505613206, 60505613207, 61703036135, 61703036250, 61703036318, 61703036322, 63323021220, 63323075010, 63323075017, 63323075020, 63323075027, 67184050801, 67184050901, 67184051001, 67457044220, 67457046850, 67457046910, 67457047610, 68083017601, 68083017701, 00004110020, 00004110150, 00054027121, 00054027223, 00093747306, 00093747489, 00179014970, 00179019570, 00378251191, 00378251278, 16714046701, 16714046801, 16729007212, 16729007329, 42291019060, 42291019112, 51079051005, 53808041101, 54868414300, 54868526000, 54868526001, 54868526002, 54868526003, 54868526005, 54868526009, 60687014994, 63759300001, 63759300101, 64980027606, 64980027712, 65162084306, 65162084406, 65162084416, 65162084450, 00009111101, 00009111102, 00009752903, 00009752904, 00009752905, 00143958301, 00143970101, 00143970201, 00703443211, 00703443281, 00703443411, 00703443481, 23155017931, 23155017932, 25021023002, 25021023005, 45963061451, 45963061455, 59923070202, 59923070205, 61703034909, 61703034916, 61703034936, 63323019302, 63323019305, 63323019352, 63323019355, 66758004801, 66758004802, 68001028425, 68001028434, 69171039801, 00002766901, 00002767801, 55513095401, 55513095501, 55513095601, 00024584001, 00024584003, 00024584101, 50242006001, 50242006101, 00143955801, 00591413054, 00781320194, 45963076257, 67457060020, 67457060130, 68152010100, 68152010201, 68152010202, 70121109901, 64842102001, 64842102002, 64842102003, 64842102501, 64842102502, 64842102503, 50419017100, 50419017103, 50419017104, 66733094823, 66733095823, 00187320447, 00187520030, 00378479106, 16110081230, 21695082940, 28105042140, 51672411802, 51672411805, 51672411806, 52549411802, 52549411805, 52549411806, 54868629300, 64370042140, 66530024940, 66530025230, 66530025830
Oxaliplatin	J9263		00024059010, 00024059120, 00069006701, 00069007001, 00069007401, 00069101001, 00703398501, 00703398601, 00781331570, 00781331780, 00781931570, 00781931780, 00955172510, 00955172720, 00955173110, 00955173320, 12516059204, 25021021120, 25021021250, 25021023310, 25021023320, 45963061153, 45963061159, 47335004640, 47335004740, 47335017640, 47335017840, 60505613206, 60505613207, 61703036135, 61703036250, 61703036318, 61703036322, 63323021220, 63323075010, 63323075017, 63323075020, 63323075027, 67184050801, 67184050901, 67184051001, 67457044220, 67457046850, 67457046910, 67457047610, 68083017601, 68083017701
